# Investigation of Study Procedures to Estimate Sensitivity and Reliability of a Virtual Physical Assessment Developed for Workplace Concussions: Method-Comparison Feasibility Study

**DOI:** 10.2196/57661

**Published:** 2024-11-27

**Authors:** Keely Barnes, Heidi Sveistrup, Mark Bayley, Mary Egan, Martin Bilodeau, Michel Rathbone, Monica Taljaard, Motahareh Karimijashni, Shawn Marshall

**Affiliations:** 1 School of Rehabilitation Sciences, Faculty of Health Sciences, University of Ottawa, 200 Lees Ave., Ottawa, ON, K1N 6N5, Canada, 1 613-612-6127; 2 Bruyère Research Institute, Ottawa, ON, Canada; 3 Acute Care Program, Ottawa Hospital Research Institute, Ottawa, ON, Canada; 4 School of Human Kinetics, Faculty of Health Sciences, University of Ottawa, Ottawa, ON, Canada; 5 Systems and Computer Engineering Technology, Carleton University, Ottawa, ON, Canada; 6 Kite Research Institute, Toronto Rehabilitation Institute, University Health Network, Toronto, ON, Canada; 7 Division of Physical Medicine and Rehabilitation, Temerty Faculty of Medicine, University of Toronto, Toronto, ON, Canada; 8 Department of Medicine, Division of Neurology, Faculty of Health Sciences, McMaster University, Hamilton, ON, Canada; 9 School of Epidemiology and Public Health, University of Ottawa, Ottawa, ON, Canada; 10 Department of Medicine, University of Ottawa, Ottawa, ON, Canada

**Keywords:** brain injury, virtual, assessment, remote, evaluation, concussion, adult, clinician review, in-person, comparison, sensitivity, reliability, acceptability survey, feasibility study, psychometric properties, vestibular/ocular motor screening, VOMS, workplace, clinician, hospital, rehabilitation center, brain, neurology, neuroscience, neurotechnology, technology, digital intervention, digital health, psychometrics, physical assessment, clinical assessment, workplace safety, mobile phone

## Abstract

**Background:**

Remote approaches to workplace concussion assessment have demonstrated value to end users. The feasibility of administering physical concussion assessment measures in a remote context has been minimally explored, and there is limited information on important psychometric properties of physical assessment measures used in remote contexts.

**Objective:**

The objectives of this feasibility study were to determine recruitment capability for a future larger-scale study aimed at determining sensitivity and reliability of the remote assessment, time required to complete study assessments, and acceptability of remote assessment to people with brain injuries and clinicians; document preliminary results of the sensitivity of the remote assessment when compared to the in-person assessment; and estimate the preliminary interrater and intrarater reliability of the remote assessments to inform procedures of a future larger-scale study that is adequately powered to reliably estimate these parameters of interest.

**Methods:**

People living with acquired brain injury attended 2 assessments (1 in-person and 1 remote) in a randomized order. The measures administered in these assessments included the finger-to-nose test; balance testing; and the Vestibular/Ocular Motor Screening (VOMS) tool, including documentation of change in symptoms and distance for near point convergence, saccades, cervical spine range of motion, and evaluation of effort. Both assessments occurred at the Ottawa Hospital Rehabilitation Center. After the assessments, a clinician different from the person who completed the original assessments then viewed and documented findings independently on the recordings of the remote assessment. The same second clinician viewed the recording again approximately 1 month following the initial observation.

**Results:**

The rate of recruitment was 61% (20/33) of people approached, with a total of 20 patient-participants included in the feasibility study. A total of 3 clinicians participated as assessors. The length of time required to complete the in-person and remote assessment procedures averaged 9 and 13 minutes, respectively. The majority of clinicians and patient-participants agreed or strongly agreed that they were confident in the findings on both in-person and remote assessments. Feedback obtained revolved around technology (eg, screen size), lighting, and fatigue of participants in the second assessment. Preliminary estimates of sensitivity of the remote assessment ranged from poor (finger-to-nose testing: 0.0) to excellent (near point convergence: 1.0). Preliminary estimates of reliability of the remote assessment ranged from poor (balance testing, saccades, and range of motion: κ=0.38‐0.49) to excellent (VOMS change in symptoms: κ=1.0).

**Conclusions:**

The results of this feasibility study indicate that our study procedures are feasible and acceptable to participants. Certain measures show promising psychometric properties (reliability and sensitivity); however, wide CIs due to the small sample size limit the ability to draw definitive conclusions. A planned follow-up study will expand on this work and include a sufficiently large sample to estimate these important properties with acceptable precision.

## Introduction

Workplace concussions impose a significant burden on the health care system, insurance providers, employers, and injured workers [1]. Conducting effective assessments after a workplace concussion is important for guiding intervention and facilitating recovery [2]. In this context, it is crucial to use measures that accurately capture deficits experienced by workers who are reporting persisting symptoms post concussion [3-5]. Ideally, an assessment should be comprehensive, consisting of measures that evaluate all domains, including physical (ie, cervical musculoskeletal and vestibulo-oculomotor function), cognitive, and socioemotional, that can be impaired by a concussion [6-8]. Furthermore, measures included in an assessment should be reliable, valid, sensitive, and possess clinical use characteristics (practical, timely, low cost, minimal equipment, etc) [3,9,10]. However, clinical concussion measures that excel in all these characteristics are limited.

Workplace concussions occur in both rural (1400 per 100,000 people [11]) and urban populations. Remote care offers an alternate approach to assessment that could be particularly beneficial for individuals living in rural areas who may experience challenges with attending in-person assessments allowing them to connect to experts that they may not have been able to access previously [12]. Remote approaches to concussion assessment have demonstrated value to end users (clinicians and patients); however, the accuracy of the findings from remote assessments has yet to be explored [13,14]. The lack of documented reliability and sensitivity properties associated with measures administered in remote assessments of people with concussions poses a barrier to the ongoing use and availability of these remote assessments in practice. Further barriers to completing remote assessments include concerns regarding safety, environmental setup, and the need for support at home [14-17]. While there are clear challenges to completing assessments, particularly physical assessments, at a distance, there are also many identified benefits [13,14].

A scoping review by O’Neil et al [18] reported a notable gap in the documentation of psychometric properties of measures administered in a remote context in people living with neurological conditions, including concussion. Agreement between in-person and remote assessments has been explored for musculoskeletal measures [19,20] and minimally explored in people living with neurological conditions [21]. Palacin-Marin et al [19] provide preliminary support for the use of remote means to administer certain measures related to low back pain such as evaluation of mobility and the visual analog scale for pain. Specifically, intraclass correlation coefficient (ICC) values for interrater and intrarater reliability ranged between 0.92 and 0.96 and the α reliability statistic was greater than .8 for the majority of measures when the remote approach was compared to the in-person approach [19]. Similarly, Cabana et al [20] examined the reliability of measures used for total knee arthroplasty. Measures, including knee range of motion, scar condition, knee joint swelling, lower limb muscle strength, timed up and go test, Tinetti gait test, and Berg balance test, evaluated using a videoconferencing platform, demonstrated good reliability (>0.80); however, levels of agreement ranged between −33% and 29% for measures of function and −20% and 8% for measures of knee range of motion [20]. Russell et al [21] compared in-person and remote physical measures for people living with Parkinson disease and reported that the physical assessment could be feasibly, reliably, and accurately completed using a telerehabilitation system. This previous work highlights a need and a value to further explore the psychometric properties of the clinical measures administered remotely for individuals with concussion.

With the COVID-19 pandemic, many clinicians have integrated remote physical assessments into their practice. Yet, there is limited research evaluating the sensitivity and reliability of the remote concussion assessment. Using a systematic approach, including focus groups [14] and working group and expert consensus [22,23], we developed a clinical assessment for individuals with a concussion. The assessment includes measures of balance, cervical spine mobility, coordination, and vestibular or ocular movements [14,22,23]. As an essential first step, prior to embarking on a full-scale study, we conducted a feasibility study. The primary feasibility objectives were to inform the methodology for a large-scale study. The feasibility measures include the rate of recruitment of both patient- and clinician-participants (willingness to participate to ensure the capability of recruiting enough participants for the large-scale study), feedback regarding the assessment approaches in the study, and preliminary information on 3 psychometric properties of the measures included in the remote assessment (interrater and intrarater reliability [24] and sensitivity [25] when administered remotely compared to in person). While this study was not designed to estimate the psychometric properties of the measures with sufficient precision, it provides some preliminary information on these metrics [26]. The study design and analysis considerations for this study were informed by Russell et al [27].

## Methods

### Ethical Considerations

Ethics approval was obtained from the Ottawa Health Sciences Network Research Ethics Board (20230311‐01H) in June 2023, followed by the Bruyère Research Ethics Board (M16-22-006) and the University of Ottawa Board of Ethics (H-06-23-9348) in June and July 2023, respectively. Patient-participants verbally consented over the telephone or provided informed consent in person. Privacy and confidentiality were maintained. Participants were provided with a parking voucher and CAD $30 (US $22) gift card following completion of participation in the study.

### Participants and Recruitment

#### Patient-Participants

People living with acquired brain injuries (ABIs) or concussions were recruited from ABI outpatient clinics publicly funded or Worker Safety and Insurance Board (WSIB) clinics based out of the Ottawa Hospital Rehabilitation Centre (TOHRC). Inpatients from the TOHRC ABI inpatient rehabilitation service were also recruited.

Eligible participants were adults aged 18 years or older who were attending a scheduled outpatient assessment or who were admitted to the ward and were under the care of one of our study clinicians. In addition to people living with concussion, people living with other forms of ABI (eg, moderate to severe traumatic brain injury and hypoxia [28]) were recruited for this study. Given that normal findings are frequently observed on the neurological examination in people with a concussion [29,30], the broader inclusion ensured that participants with identifiable positive findings on the neurological examination were represented in the sample. Participants unable to speak English or French and unable to complete both the in-person and remote assessment procedures were excluded.

The electronic medical records of patients attending in-person appointments and the list of patients admitted to the ward were screened to identify eligible individuals who were then approached via telephone (outpatients) or face-to-face (inpatients) to discuss possible participation in the study.

No sample size calculation was conducted for the feasibility study. Instead, we planned to recruit participants for a period of 5 months based on logistical considerations. We plan to include the data from the feasibility study in the future larger-scale study, assuming the protocol does not need to be modified between the feasibility and definitive study.

#### Clinician-Participants

Clinicians who were employed at TOHRC and were actively completing ABI assessments were recruited to participate. Assessors were responsible for completing the study assessments and observing and rating recordings of completed assessments. Eligible clinicians were approached via telephone and consented over the telephone.

#### Training

The Virtual Concussion Exam Manual [31] was adapted and reviewed by all clinicians prior to their participation in the study. The manual contains clear instructions on administering clinical measures remotely. Clinicians were encouraged to consult the adapted manual for guidance while conducting study assessments.

### In-Person and Remote Assessments

Each patient-participant completed both the in-person and remote assessment at TOHRC on the same day. The assessments were conducted during a scheduled in-person appointment for outpatients, or a specific appointment set up for inpatients. The 2 assessments were separated by a brief rest period [32] with additional rest time provided until the participant could complete the remainder of the measures.

The order of the assessments (in-person and remote) was randomized and counter-balanced to ensure an equal number of participants completed the in-person and remote assessments first. Randomization occurred through REDCap (Research Electronic Data Capture; Vanderbilt University) using a random numbers table. The rationale was to equalize the influence of fatigue and learning effects on the subsequent assessment. The assessments consisted of the same clinical measures (Table 1).

For the remote assessment, the patient-participant was in a separate room from the assessor. All patient-participants used the same computer and received technical support from a research team member if needed. Safety precautions during the remote assessment included (1) the presence of a research team member in the room throughout the remote assessments and (2) positioning patient-participants in front of a wall, chair, or bed during balance testing. All remote assessments used a Dell Vostro 3520 Laptop (with a 15.6-inch display) running the Microsoft Teams platform (for the patient-participants). Assessors used desktop computers. The remote assessments were audio-video recorded for later review and evaluation by a different clinician. For the in-person assessment, a research team member was present in the room to record the assessment using the same laptop as the remote assessment. Safety precautions were provided by the assessing clinician.

**Table 1.** Outline of measures included in the remote assessment, clinical decision for each measure, and guideline for clinical decision.

Measure	Clinical decision	Guideline for decision	Reference
Finger-to-nose test	Normal or abnormal	Abnormality considered hesitation, tremor, and undershooting or overshooting	[33]
Balance testing (feet together, single leg stance, and tandem stance) for 20 seconds with eyes open and closed	Normal or abnormal	Abnormality considered inability to hold the position for 20 seconds	[31]
Saccades	Normal or abnormal	Abnormality determined by saccade speed, accuracy, initiation, intrusions or oscillations, and range of motion and conjugacy	[34]
VOMS^a^—change in symptoms	Changes in symptoms documented following completion of each component	Change greater than or equal to 2 points out of 10 was considered abnormal	[35]
VOMS—near point convergence	Distance documented	A distance greater than or equal to 5 cm was considered abnormal	[35]
Cervical spine range of motion	Estimated angles for cervical flexion, extension, lateral flexion, and rotation recorded	Values were compared to pooled norms for healthy individuals aged 20 to 59 years old documented in a recent systematic review to identify abnormality: flexion=50‐72°, extension=58‐77°, lateral flexion=37‐47°, and rotation=67‐81°	[36]

a VOMS: Vestibular/Ocular Motor Screening.

### Observation of Recordings of Assessments

**Figure 1.**
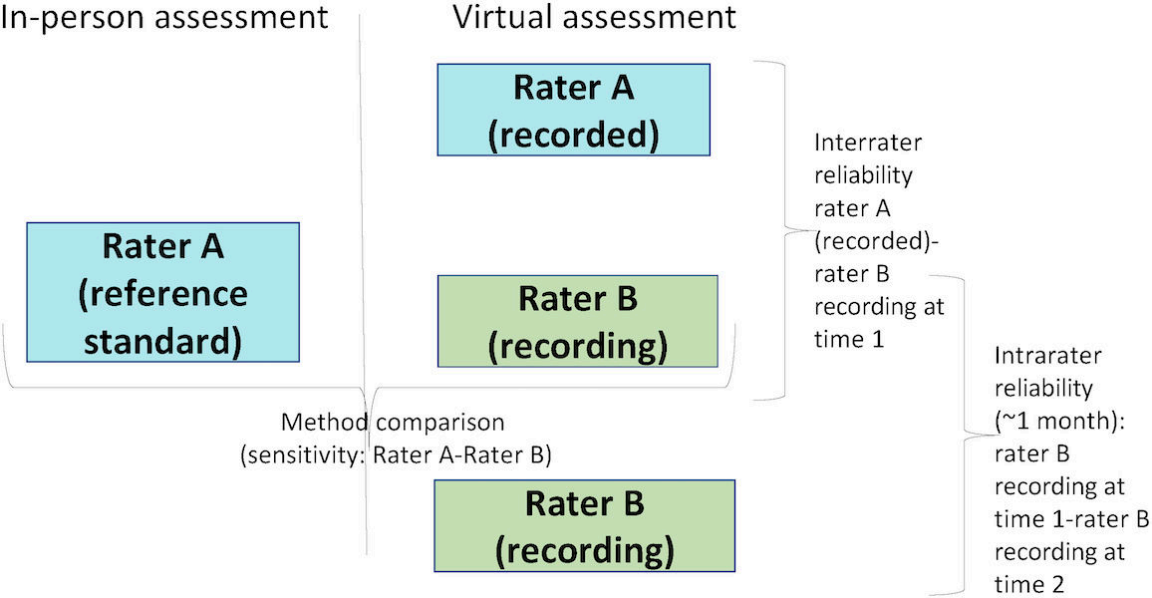
Assessment procedures modified from Russell et al [27].

Two assessors (rater A and rater B) documented clinical findings for each patient-participant. Rater A completed the in-person and synchronous remote assessments. Rater B documented findings asynchronously from audio-video recordings of the remote assessment at 2 time points separated by approximately 1 month. Figure 1 outlines the study assessment procedures.

### Feedback

Feedback was obtained from both clinician- and patient-participants using a feedback form, following completion of the assessments. Participants answered questions related to the environmental setup of the remote assessment, confidence in the assessment findings, perceived similarity between the 2 approaches, and provided any additional feedback on the form.

### Analysis

#### Rate of Recruitment and Time Required to Complete Study Procedures

Descriptive data are provided for an average number of participants recruited per month, rate of recruitment, and average and range of time required for in-person and remote assessments separately.

#### Participant Characteristics, Feedback, and Confidence Ratings

Participant characteristics (sex, age, injury information, etc) were analyzed descriptively. Feedback obtained following the completion of study assessments and perceived confidence ratings in findings on study assessments for both clinician- and patient-participants were summarized narratively.

#### Reliability and Sensitivity

Quantitative data analyses were completed using IBM SPSS (version 28, IBM Corp). Interrater reliability was determined by comparing the results documented by rater A (remote assessment) with those documented by rater B (remote assessment at time 1). Intrarater reliability was determined by comparing the results from rater B at time 1 versus time 2. All measures were coded into binary categories (abnormal vs normal; see Table 1). Some measures had single items (Vestibular/Ocular Motor Screening [VOMS] change in symptoms, VOMS near point convergence, and saccades), whereas some measures (balance testing, cervical spine range of motion, and finger-to-nose testing) included multiple items where each was rated as 0 or 1 and results were summed. For both interrater and intrarater reliability, unweighted κ statistics were calculated. κ is calculated by dividing the difference between the observed agreement (proportion of times raters agreed) and expected agreement by chance (agreement that would occur by random chance) by 1 minus the expected agreement by chance. κ values between 0 and 1.0 were documented where values closer to 0 indicate poor reliability and values closer to 1.0 indicate perfect agreement [37]. The 95% CIs were calculated manually by multiplying the standard errors obtained from SPSS by the *z* score statistic 1.96 and adding and subtracting that value to or from the κ values to obtain the upper and lower bounds [38].

The sensitivity of the remote assessment was determined by comparing the results documented by the clinician who observed and rated the recording of the remote assessment (rater B at time 1) with the results documented by the clinician conducting the in-person assessment (rater A, reference standard) [27]. The value reflects the proportion of people identified as impaired on the in-person measure who were identified as impaired on the remote measure [25]. Sensitivity is calculated by dividing the true positives (identified correctly as impaired on the in-person and remote assessments) by the sum of the true positives and false negatives (the remote assessment failed to identify as impaired, but the in-person assessment correctly identified as impaired). The 95% CIs were calculated using the online VassarStats Clinical Calculator [39].

#### Adverse Events

Adverse events, including the severity and type, were monitored. Significant worsening of symptoms requiring medical attention, such as an emergency department visit or a new injury, was considered an adverse event [40].

## Results

### Participant Characteristics

A total of 20 patient-participants completed both the in-person and remote assessments (see Table 2). In it, 15 (75%) participants were female. Most participants were working at the time of their assessment. Most participants reported limitations in functional abilities and perceived their mental health as fair to poor. When considering the criteria outlined regarding the identification of abnormality on each measure, 18 out of the 20 participants were abnormal on at least 1 of the measures in the in-person assessment.

The injury characteristics of the 20 patient-participants are presented in Table 3. A total of 14 (70%) participants sustained a concussion, and the remainder sustained a moderate to severe traumatic brain injury or other form of ABI, with most injuries occurring outside of the workplace context.

Most participants used technology daily and the majority rarely needed assistance when using technology (Table 4).

Three clinicians participated as assessors. Two assessors (male and female), acting as rater A, were a physiatrist and a physician assistant. The third clinician (a male), acting as rater B, was a physiatrist and observed the recordings of the assessments. These clinicians typically assess more than 50 patients with ABI annually and reported feeling confident in their ability to complete the in-person and remote assessments.

**Table 2.** Demographic characteristics (N=20).

		Values
Age (years), range		21‐58
**Sex, n (%)**		
	Female	15 (75)
	Male	5 (25)
**Gender, n (%)**		
	Woman	15 (75)
	Man	5 (25)
**Ethnicity, n (%)**		
	White	15 (75)
	Black	1 (5)
	Arab	1 (5)
	Southeast Asian (eg, Vietnamese, Cambodian, Malaysian, and Laotian)	1 (5)
	West Asian (eg, Iranian and Afghan)	1 (5)
	First Nation or Indigenous	1 (5)
**Highest educational attainment, n (%)**		
	Secondary (high) school diploma or equivalent	8 (40)
	Postsecondary certificate, diploma, or degree	12 (60)
**Current work status, n (%)**		
	Off work	7 (35)
	Modified return to work, same preinjury occupation	6 (30)
	Full return to work, same preinjury occupation	4 (20)
	Full return to work, different occupation	2 (10)
	First time working	1 (5)
**Functional limitations, n (%)**		
** Moderate activities**		
	Yes, limited a lot	3 (15)
	Yes, limited a little	10 (50)
	No, not limited at all	7 (35)
**Climbing stairs**		
	Yes, limited a lot	3 (15)
	Yes, limited a little	8 (40)
	No, not limited at all	9 (45)
**Perceived mental health, n (%)**		
	Excellent or very good	2 (20)
	Good	6 (30)
	Fair or poor	12 (60)

**Table 3.** Injury information (N=20).

Characteristics		Values
**Diagnosis, n (%)**		
	Other acquired brain injury	6 (30)
	Mild traumatic brain injury or concussion	14 (70)
**Mechanism of injury, n (%)**		
	Work	6 (30)
	Motor vehicle accident	4 (20)
	Assault	2 (10)
	Fall or hit head at home	4 (20)
	Sport	2 (10)
	Poisoning	1 (5)
	Encephalitis	1 (5)
**Date of injury, n (%)**		
	<6 months ago	3 (15)
	6 months to <1 year ago	3 (15)
	1 to <2 years ago	7 (35)
	2 to <3 years ago	3 (15)
	>3 years ago	4 (20)

**Table 4.** Remote assessment and technology experience (N=20).

Characteristics		Values
**Previously attended remote assessment, n (%)**		
	Yes	11 (55)
	No	9 (45)
**If yes, number attended, n (%)**		
	<5	8 (40)
	5‐10	1 (5)
	>10	1 (5)
	Unsure	1 (5)
**Distance located from the rehabilitation center, n (%)**		
	<30 minutes	9 (45)
	30‐60 minutes	8 (40)
	>60 minutes	2 (10)
	N/A^a^—no home	1 (5)
**Technology available for remote assessment, n (%)**		
	Computer	2 (10)
	Laptop	8 (40)
	Smartphone	2 (10)
	Multiple devices (iPad, smartphone, and computer)	7 (35)
	None	1 (5)
**Use of technology, n (%)**		
	Weekly	3 (15)
	Daily	17 (85)
**Assistance needed during use of technology, n (%)**		
	Never	7 (35)
	Rarely	9 (45)
	Monthly	2 (10)
	Weekly	1 (5)
	Daily	1 (5)

a N/A: not applicable.

### Rate of Recruitment

We experienced challenges recruiting clinicians willing to conduct the study assessments, and clinicians’ scheduling conflicts posed difficulties with patient-participant recruitment; therefore, the involvement of additional professionals, such as physiotherapists, will be needed to support the large-scale study. We recruited, on average, 1 patient-participant per week at TOHRC. A total of 38 potential participants were identified. In it, 7 could not be reached. Of the potential participants reached, 6 (23%) declined, primarily due to concerns that the multiple assessments would make their symptoms worse. Our rate of recruitment was, therefore, 20/33 (61%) of people approached and 20/26 (77%) of people reached. Given the recruitment rate, at 1 center, we anticipate being able to approach approximately 6 participants per month. With the anticipated recruitment rate of 61% over 5 months, we can feasibly recruit 20 participants, which would mean the future large study with a target sample size of 60 [41] would require 15 months to complete recruitment.

### Length of Time Required to Complete Study Assessments

The time required to complete the in-person assessment ranged from 5 to 13 minutes and averaged 9 minutes. The time required to complete the remote assessment ranged from 7 to 26 minutes and averaged 13 minutes.

### Feedback

#### Clinician-Participants

##### Perceived Similarity of Remote and In-Person Assessments

The 2 assessors (raters A) believed that they obtained similar information from both the in-person and remote assessment in a majority 16/20 (80%) of cases. On 4 occasions, the assessors reported that the patient-participant had fatigue and experienced heightened symptoms during the second assessment; however, on 2 of these 4 occasions, the assessor still believed that comparable findings were obtained even with the exacerbation. For example, 1 clinician reported that the findings were “comparable, but patient fatigued and became more symptomatic,” and another reported an *“*increase in symptoms during the second assessment.” On 2 occasions, the assessor reported that they did not believe similar information was obtained, as one of the patient-participants felt more comfortable in person and the other patient-participant was able to follow directions better in person, reporting that the participant “had an easier time with directions in-person.”

##### Challenges

One assessor expressed some issues with remote tests due to dark lighting in the assessment rooms when blinds were open, and patient-participants were positioned in front of windows, reporting “lighting in background, lost sight of pen.” Another assessor noted audio issues (“sound delay”) during the remote assessment with sound going in and out, forcing the assessor and patient-participant to repeat statements. Technical challenges related to internet connectivity were experienced on 2 occasions, with reports of “internet freezing in exam room” and “initial connection slow*.*”

### Patient-Participants

#### Perceived Similarity of Remote and In-Person Assessment Findings

Most (12/20, 60%) of the patient-participants believed that similar findings were obtained in both the in-person and remote assessments. One patient-participant was unsure about the similarity of findings, reporting “measurement wise, I can’t say.” Three patient-participants mentioned that ocular tests may have been easier for the clinician to observe in person. For example, 1 participant reported that “there might have been a difference in visual tests” and another reported that “I think eye movements are more easy to observe in-person.” One patient-participant reported that more relevant clinical data were obtained in person, 1 reported that their concussion symptoms were worse remotely due to screen exposure, 1 expressed doubt that the remote assessment would have been sufficient for their initial assessment, and 1 perceived that similar information was not obtained as the conversation was easier in person.

#### Challenges

Patient-participants expressed minimal concerns regarding the environmental setup of the remote assessment. One patient-participant reported that it would be helpful to see the whole body of the clinician for demonstrations of measures or to observe a photo of the measure on the screen beforehand. Another noted that while the setup was adequate, space was limited for one of the measures that required her to rotate her body. Technical support was required for all participants to manage camera angles during remote completion of balance testing. One participant highlighted the value of having someone physically present to troubleshoot any camera angle challenges. Two participants suggested that a larger screen size would be beneficial, with 1 reporting that “the screen was a bit small and needed to be adjusted to capture all my movements” and another reported that “a bit larger screen might be better for evaluation to see eye movements.” Finally, 4 patient-participants reported that the lighting was an issue for both the in-person and remote assessment due to light sensitivity associated with the concussion, whereas assessors commented on lighting in relation to the visibility of the patient-participant during the remote assessment. For example, 1 patient-participant reported that “lighting would be great on a dimmer” and another reported that the assessments were “a bit bright with the lights on*.*”

### Confidence Ratings

The assessors expressed high confidence in their findings on the in-person (20/20, 100%) and remote (19/20, 95%) assessments. Only on 1 occasion was an assessor “neutral” in terms of their confidence in their findings on the remote assessment.

Most patient-participants agreed or strongly agreed that they felt confident in their assessors’ findings on the in-person (19/20, 95%) and remote (15/20, 75%) assessments. Two patient-participants did not feel confident and 2 were “neutral” in their confidence levels on the remote assessment. One participant did not feel confident in both the in-person and remote assessments.

### Preliminary Information on Sensitivity of the Remote Assessment Compared to the In-Person Assessment

The preliminary sensitivity of the remote compared to in-person administration of the measures ranged from 0.0 to 1.0 (Table 5). This suggests poor (finger-to-nose testing) to excellent (near point convergence) ability to detect deficits in the remote assessment when deficits are truly present (based on the reference standard, which is the in-person assessment). On 2 occasions, evaluation of saccades was not documented by the assessor in error. On 1 occasion, the VOMS was not completed due to the patient’s inability to complete the measure because of aggravation of symptoms. No abnormality was documented for effort on both the in-person and remote assessment and, therefore, sensitivity could not be computed.

**Table 5.** Sensitivity of the remote assessment compared to the in-person assessment.

Measure	Values, n	Sensitivity (95% CI)	
**Cervical spine**			
ROM^a^	20	0.33 (0.09-0.69)	
**Vestibular**			
Balance—eyes open and closed: feet together, single leg stance, and tandem stance	20	0.94 (0.69-1.0)	
VOMS^b^—change in symptoms	19	0.92 (0.60-0.99)	
VOMS-NPC^c^	19	1.0 (0.70-1.0)	
**Neurological examination**			
Finger-to-nose	20	0.0 (0.0-0.60)	
**Oculomotor**			
Saccades	18	0.50 (0.09-0.91)	
**Effort**			
Optimal effort	20	—^d^	

a ROM: range of motion.

b VOMS: Vestibular/Ocular Motor Screening.

c NPC: near point convergence.

d Statistic could not be computed, as the values documented by both assessors are constant for this measure (all normal findings reported).

### Preliminary Information on Interrater and Intrarater Reliability

Table 6 presents the preliminary information on the interrater and intrarater reliability of the measures when administered remotely. Cohen κ values for interrater reliability ranged from poor for balance testing (0.38), range of motion (0.47), and saccades (0.49) to excellent (1.0) for VOMS change in symptoms. The intrarater reliability ranged from poor (0.44) for the evaluation of saccades to very good (0.89) for VOMS change in symptoms.

**Table 6.** Interrater and intrarater reliability of the measures when administered remotely.

Measure	Intrarater reliability			Interrater reliability		
	Value, n	Cohen κ (95% CI)		Value, n	Cohen κ (95% CI)	
**Cervical spine**						
ROM^a^	20	0.69 (0.29 to 1.0)		20	0.47 (0.04 to 0.90)	
**Vestibular**						
Balance testing—eyes open and closed: feet together, single leg stance, and tandem stance	20	0.61 (0.11 to 1.0)		20	0.38 (−0.09 to 0.86)	
VOMS^b^—Change in symptoms	20	0.89 (0.67 to 1.0)		20	1.0 (1.0 to 1.0)	
VOMS-NPC^c^	20	0.76 (0.47 to 1.0)		20	0.67 (0.34 to 0.99)	
**Neurological examination**						
Finger-to-nose	20	—^d^		20	—	
**Oculomotor **						
Saccades ** **	20	0.44 (−0.2 to 1.0)		18	0.49 (0.04 to 0.94)	
**Effort **						
Optimal effort	20	—		20	—	

a ROM: range of motion.

b VOMS: Vestibular/Ocular Motor Screening.

c NPC: near point convergence.

d Statistic could not be computed, as the values documented by the second assessor are constant for these measures (all normal findings reported).

### Adverse Events

Most (17/20, 85%) participants reported an increase in preexisting brain injury symptoms, such as increased headaches, dizziness, and nausea, during the VOMS measure; however, this was true for both the in-person and remote assessments.

## Discussion

### Principal Findings

This is one of the first studies to examine the preliminary psychometric property information of concussion assessment using remote approaches. According to Montes et al [42], a systematic approach to remote assessment development is essential, with established in-person measures serving as a basis for comparison or as a reference standard. Taking this into consideration, we first carried out reviews of the psychometric properties of potential measures to help select the measures to be included in our remote assessment [14,22,23]. In this study, we report on the acceptance, feasibility, and preliminary psychometric properties of these assessments to provide initial evidence of the remote assessment and inform a larger study of remote assessment for concussions.

The rate of recruitment is a critical indicator of the success of a future large-scale study. Our moderate rate of recruitment indicates that there is sufficient interest among people with ABI to participate in a remote assessment-based study; however, additional strategies may be needed in order to increase our ability to reach potential participants. Although patient-participants showed a moderate willingness to participate, we did experience more difficulties recruiting males and recruiting people with other forms of ABI when compared to the recruitment of people with concussions. This was mainly because the practice of one of our assessors only included people with concussions (and not other forms of ABI). We experienced some difficulty recruiting people with known abnormality on specific tests, particularly those with abnormal coordination, as people with concussion typically have normal coordination and recruitment of people with concussion was easier due to the nature of the practice of our recruited assessors. The rate of patient recruitment appeared to be influenced by clinical status (concerns regarding aggravation of symptoms due to the nature of the measures and the need to complete the measures twice). Hunt et al [43] reported that stakeholder engagement in concussion research may be inhibited by injury-related factors, personal deterrents (vulnerability and fear), and environmental barriers. Concussion symptoms, including physical, cognitive, and emotional, were identified as a barrier to involvement by patient-participants [43].

The length of time required to complete our study procedures is another essential aspect of evaluating the feasibility of completing a large-scale study and participant burden. Factors influencing the time required to complete the study assessments included the participant’s ability to follow instructions, participant symptom aggravation, and technology issues (internet speed). The average time required to complete our remote assessment was longer than the in-person assessment, which is contrary to findings reported by Tran et al [44] who noted that remote visits tend to be similar in length when compared to in-person visits. However, adaptation of certain measures to remote environments, such as the VOMS, has been previously reported to take time and practice [45], which may have contributed to the additional time required to complete the remote assessment. While the duration of the remote assessment was longer than the in-person one, the reduced travel time required to attend an in-person visit highlights the convenience associated with remote assessments from the patient-perspective [46,47], although increased clinician time required may be a concern [14].

Additionally, having technical support with camera angles may have positively impacted the experience of the participants with the remote assessment in this feasibility study. A research team member was present to aid with moving the laptop to improve the visibility of the patient for the clinician, close blinds, and troubleshoot internet issues. Ownsworth et al [48] reported that ongoing access to support could improve user-friendliness and facilitate the use of remote care. Further, there is a need for reliable and high-quality videoconferencing technology, which could present a challenge in practice due to variable accessibility and cost [49]. To improve the issue associated with lighting, which was expressed by participants, it is recommended that blinds are closed when completing remote assessments and lights are bright enough for clinicians to observe the individual on screen but are manageable for the individual in terms of limiting symptoms aggravation. To improve visibility, a blank backdrop is recommended along with appropriate positioning of the camera at eye level [50].

Participants perceived the environmental setup of the remote assessment to be adequate. The feedback obtained highlighted the advantages of remote assessments over traditional in-person assessments, which is consistent with the literature, including eliminating the need to drive to the assessment center, and the ability to control the environment better at home, such as having the capacity to dim lights [46,51,52]. However, home can present challenges as well [53]. The remote assessments for this study were all conducted using the same laptop and within the same setting, which may have positively impacted the experiences of the participants. The home environment may present unique challenges, such as distractions and variable screen sizes, which in turn may impact outcomes on the assessment [54]. It is recommended to develop a plan and schedule for these assessments to minimize such distractions in the home setting [54]. Further, 1 participant in our study was homeless and was admitted as an inpatient, and therefore, would not have access to needed technology in a home environment.

When remote assessments are implemented in practice, it is important to ensure that findings obtained through the remote assessment are comparable to those obtained through in-person approaches [27]. For the most part, the clinician- and patient-participants in this feasibility study perceived that though findings were similar in the in-person and remote assessment. This perception of congruence is supported by objective data obtained by Vargas et al [55] who examined the feasibility of remote assessment of concussion using a telemedicine robot in which a neurologist remotely assessed injured athletes simultaneously to sideline provider in-person assessments. Vargas et al [55] provide preliminary information on the strong level of agreement (within 3 seconds or points 100% of the time) between findings documented by the in-person sideline provider and the remote neurologist on specific concussion measures (Standardized Assessment of Concussion [a cognitive test], modified Balance Error Scoring System [a balance test], and King-Devick test [a saccadic eye movement test]) [55]. While the perception of congruence was high, participants, both assessors and patients, were more often more confident in the findings of the in-person assessment when compared to the remote assessment. These findings are in line with Gilbert et al [56], who noted that clinicians and patients were satisfied with remote consultations; however, in-person consultations are still preferred (outside the COVID-19 pandemic).

In-person measures should have acceptable reliability properties for method-comparison studies to have meaningful results [27]. It is, therefore, recommended to determine the reliability properties of the remote assessment as part of the method-comparison study. Sensitivity metrics of the measures included in the remote assessment are also of interest, as clinical assessment findings are relied upon to detect deficits and guide intervention [2]. The preliminary investigation of reliability and sensitivity in this study appears to vary when compared to previously reported in-person values. Reliability values for in-person administration of the measures range from moderate (test-retest reliability of the single leg stance, with a κ of 0.43 [57]), to excellent (within-tester reliability of cervical spine range of motion with an ICC of 0.90 [58]). The sensitivity metrics associated with in-person administration of the measures range from moderate (0.45 for balance testing in people with traumatic brain injury [59]) to excellent (96% for the VOMS assessed in people with concussion [58]). The findings of this feasibility study indicate that the evaluation of finger-to-nose testing, saccades, and cervical spine range of motion appear to have poorer properties associated with remote administration when compared to in-person metrics, which have documented sensitivities of 71% [59], 64%‐77% [60], and 86%‐95% [61], respectively.

Consistent with the literature, the lack of physical presence in the remote assessment may have been associated with an inability to gain a full understanding of the patient’s status in these measures and subtle abnormalities such as those observed during oculomotor assessments may have been more difficult to capture through videoconferencing [62,63], which was also subjectively acknowledged by patient-participants in this feasibility study. Documentation of psychometric properties of measures administered in both in-person and remote contexts is required to support the hybrid approach to care, which integrates both in-person and remote interactions with clinicians. This approach is desired by both clinicians and people living with concussions [13,14].

The data obtained in this study suggest that there are specific measures, such as the evaluation of saccades, cervical spine range of motion, and finger-to-nose testing that are more difficult to administer remotely and, therefore, a high level of care in delivery should be considered to increase reliability and sensitivity. Further development and research are, therefore, needed in this area to determine how best to administer these measures remotely. This could include using advanced technologies, improving training of clinicians, or improving administration instructions [50], all of which will be considered for the large-scale study. Best practices for administering these measures need to be identified, and strategies to overcome limitations posed by the remote environment should be explored.

### Strengths and Limitations

A strength of this study includes the use of technology and software that are commonly used to complete remote assessments at TOHRC. This decision was made to mimic as best as possible the current clinical approach to remote assessments. Further, we expanded the inclusion criteria to include participants with various forms of ABI, which ensured the inclusion of individuals with identifiable positive findings. This approach not only facilitated recruitment but also enhanced the generalizability of our findings.

Our study also has several limitations. First, this study was carried out in a controlled environment to standardize as many aspects of the remote assessment as possible. Thus, we did not test the remote assessment in real-world settings such as with people present in remote or rural regions, where factors such as home environments, technology used, internet, and so forth, may have influenced reliability. Second, due to an inability to complete the assessments more than twice, we were unable to establish the reliability properties of the in-person assessment and relied on the literature for the in-person properties needed for comparisons, which will also be required for the large-scale study. Third, it was not feasible for 2 different clinicians to conduct the initial in-person and remote assessments in our clinical environment due to scheduling constraints, so the same clinician completed both initial study assessments. In an attempt to mitigate potential bias and in line with best practices for method-comparison studies comparing in-person and remote approaches [27], we randomized the order of the study assessments and compared the in-person assessment conducted by rater A with the findings of the observed recording completed by rater B; however, this approach is limited in that rater differences may have impacted the findings. Finally, the widths of the CIs reported in this feasibility study are extremely wide (due to the small sample size) and, therefore, there is little conclusion one can draw from the psychometric property estimates. The small sample size further limits the generalizability of the study findings. More data and extensive study are needed to definitively establish the reliability and sensitivity properties associated with the remote concussion assessment.

### Conclusions

For clinical measures to be confidently used by clinicians in remote care practice, comparisons need to be made to their in-person counterparts. Given the width of the CIs, little can be concluded regarding the sensitivity of the concussion measures administered remotely, when compared to in-person administration, and the reliability of those measures. However, this feasibility study documents the time needed to complete components of a concussion assessment remotely and confirms the probable safety of the assessment, with no adverse events specific to the remote assessment documented. The findings of this feasibility study provide a foundation and will inform processes for a planned follow-up study that contains an adequate sample size to estimate the psychometric properties with adequate precision. Future work should expand on this foundation through the exploration of the impacts of home environments on remote concussion assessment outcomes and through the investigation of additional relevant psychometric properties, such as responsiveness.

## Abbreviations

ABI: acquired brain injury
ICC: intraclass correlation coefficient
REDCap: Research Electronic Data Capture
TOHRC: Ottawa Hospital Rehabilitation Centre
VOMS: Vestibular/Ocular Motor Screening
WSIB: Worker Safety and Insurance Board
